# Short-Term Outcomes of First Metatarsophalangeal Arthroplasty Using the Silktoe Double-Stemmed Silicone Implant

**DOI:** 10.3390/diagnostics15111349

**Published:** 2025-05-27

**Authors:** Stefano Fieschi, Costanza Redaelli, Anita Fazzini

**Affiliations:** 1 Department of Orthopaedic Surgery, Casa di Cura Villa Berica, Via Giuseppe Capparozzo, 10, 36100 Vicenza, Italy; 2 Department of Orthopaedic Surgery, Casa di Cura S. Francesco, Via IV Novembre, 7, 24128 Bergamo, Italy; costanzaredaelli@gmail.com; 3 Department of BRM Extremities, Via Privata ai Campi, 7, 23862 Civate, Italy

**Keywords:** hallux rigidus, metatarsophalangeal joint, metatarsophalangeal arthroplasty, silicone implants

## Abstract

**Background**: Hallux rigidus is a painful and degenerative pathology of the first metatarsophalangeal (MTP1) joint. In severe cases it is usually appropriate to consider arthrodesis or arthroplasty of the joint. Arthrodesis represents the gold standard, but arthroplasty allows patients to recover mobility. Although arthroplasty has slightly inferior functional results to arthrodesis, it has shown very good results in terms of joint mobility, patient satisfaction and pain reduction. The goal of the present study was to evaluate short-term outcomes of patients that received a third-generation double-stemmed MTP1 implant manufactured from high-performance silicon. **Methods**: In this retrospective study the authors analyzed data of 37 patients who underwent MPT1 joint arthroplasty with Silktoe^TM^ double-stemmed implant using the R 4.2.2 software (R Foundation for Statistical Computing, Vienna, Austria). The indications were hallux rigidus of grade III and grade IV (81.1%), hallux rigidus plus valgus (10.8%), painful or unstable joint following previous surgery (5.4%) and hallux rigidus due to gout (2.7%). Data were collected during routine visits at 1 and 6 months and 1 and 2 years. **Results**: There were no intraoperative and postoperative complications. There were no revisions or reoperations at a follow-up of two years. The American Orthopaedic Foot and Ankle Society-Hallux Metatarsophalangeal-Interphalangeal Scale (AOFAS-HMI) score was 94.6 ± 7.6 (median, 100; range, 70–100) and the Visual Analogue Scale (VAS) score was 0.4 ± 0.80 (median, 0; range, 0–3.5) at the final follow-up. **Conclusions**: The data from this study presented excellent short-term results for patients who received an arthroplasty of the MTP1 joint using a third-generation double-stemmed spacer made of high-performance silicone. For all patients who received the Silktoe^TM^ implant, AOFAS-HMI scores of 94.6 and VAS scores of 0.4 were obtained at a two-year follow-up. These values were in line with results reported in the literature for similar devices.

## 1. Introduction

The first metatarsophalangeal (MTP1) joint holds most of the body weight and plays a main role during the gait propulsion movement. The MTP1 joint mainly allows movements of dorsi flexion, bending dorsally, and plantar flexion, bending plantarly, but it also performs rotational movements and some abduction and adduction movements [1].

Hallux rigidus is a painful and degenerative pathology of the MTP1 joint that is quite common in the adult population (10%), with a higher incidence in the female population. The average age of occurrence of symptoms is 43 years, while the average age at which patients undergo surgery is 50 years. In 95% of cases, the condition arises bilaterally; the remaining cases are related to traumatic events [2]. It causes pain and stiffness of the affected joint to the point of causing difficulty in daily activities and an alteration of the load distribution on the foot and the patient’s gait [3,4]. In the more advanced stages of hallux rigidus, there is often the formation of bony prominences, called osteophytes, in the periarticular area. Such osteophytes further restrict joint motion [1,4]. Today, the most commonly used classification of the severity of this pathology is the Coughlin and Shurnas classification, which divides hallux rigidus into five stages using both clinical and radiographic parameters [4].

Mild or moderate cases may benefit from conservative therapy or less invasive surgical treatments, such as cheilectomy, osteotomy and joint distraction, while in more severe cases it is usually appropriate to consider arthrodesis or arthroplasty of the MTP1 joint [5]. Cheilectomy involves removal of all asperities in the joint responsible for reduced motion. However, the recurrence of dorsal osteophyte formation is about 30%, and such surgery is reserved exclusively for cases of mild and medium hallux rigidus [4,5,6]. For these cases, osteotomies of the first metatarsal and/or proximal phalanx can also be performed, but in general, the use of osteotomy for the management of hallux rigidus is not adequately supported in the literature [5]. Joint distraction of the MTP1 joint can be used in painful joints with limited mobility [4].

Arthrodesis is still considered the gold standard for severe cases of hallux rigidus; however, this technique results in a total loss of mobility of the joint [7,8,9,10,11,12,13,14]. The predictable and safe outcomes of arthrodesis make it a viable alternative for the revision of other failed procedures. Arthroplasty, on the other hand, is associated with a more uncertain outcome, but allows patients to recover mobility and reduce pain [14,15,16].

The arthroplasty developed by Keller involves resection of the base of the proximal phalanx to decompress the joint and create space to increase range of motion. Keller’s procedure is recommended as an alternative to arthrodesis only in patients over 70 years of age who are not very active [4,5]. Modifications of the Keller procedure combined with interposition arthroplasty techniques have also been suggested, particularly in young and active patients who refuse MTP1 fusion [13,17].

Another alternative is prosthetic arthroplasty. Both metal and silicone hemi-implants and implants are available for the MTP1 joint. Although arthroplasty has slightly inferior functional results to arthrodesis, it has shown very good results in terms of joint mobility, patient satisfaction and pain reduction, especially with double-stemmed silicone devices and metal hemi-implants. Indeed, a meta-analysis of 47 papers identified four generations of spacers for the MTP1 joint and classified them into five categories according to the material used (silicone, metal and ceramic) and design (partial or double-stemmed implants), reporting high patient satisfaction with double-stemmed silicone implants and metal hemi-implants [18,19,20]. An alternative solution is the polyvinyl alcohol (PVA) hydrogel implant, which, once implanted, acts as a spacer between the first metatarsal and the base of the proximal phalanx. However, there are high rates of implant failure and loss of quality of life. In addition, a large bone deficit remains after device removal, making it necessary to consider autograft or structural allograft to restore the length of the first radius at revision [2,21].

The first attempts at implant arthroplasty of the MTP1 joint date back to the 1950s when Swanson and Seeburger tried unsuccessfully to design prostheses of the metatarsal head [4]. It was in the late 1970s that Swanson proposed the use of the double-stemmed silicone spacer, which had wide initial acceptance due to the good results obtained in the short term [22]. However, the expectations placed on these implants were disappointed by the occurrence of a number of medium- and long-term complications, such as synovitis, osteolysis, lymphadenopathy and device fractures, reported by Vanore et al. [3,4,19].

In the 1980s, there was an evolution in silicone implants, which led to the development of the second-generation MTP1 implants, modified to better respect the anatomy of the implant site: LaPorta’s ‘Silastic’ implant and Lawrence ‘Siflex’ implant. Meanwhile, Swanson modified the implant by shortening the stems and adding titanium grommets to create a bone–prosthesis interface and repair the silicone from abrasion related to contact with the resected bone surfaces [19,20].

The silicone implants currently available are ‘third-generation’ implants, designed based on the specific anatomy of the MTP1 joint and made of silicone elastomers of varying densities, which have better tensile and tear resistance properties [3,4]. These devices have been in use since 1997 and have been confirmed to be viable options for the treatment of hallux rigidus, although they are associated with some side effects. The use of grommets, which protect the silicone spacer from abrasion and help to improve its strength, are also recommended [3,4,23].

The results of implantation of these devices are variable, being related to several factors: type of implant, patient population, age and functional ability of the patient, length of follow-up and presence of other foot deformities. The main complication reported is fracture of the device; however, in many cases this is not associated with implant failure or patient dissatisfaction. Other possible complications are related to device wear and the release of small silicone particles, resulting in synovitis [2,3,6].

The goal of the present study was to report and evaluate short-term outcomes of a group of patients that received a third-generation double-stemmed MTP1 implant manufactured from high-performance silicone.

## 2. Materials and Methods

### 2.1. Study Design

The authors analyzed the reports of 59 patients (59 feet) who underwent MPT1 joint arthroplasty between January 2018 and November 2022 performed by the same physician (SF). All patients received the Silktoe^TM^ double-stemmed MPT1 implant (BRM Extremities, Civate, Italy).

Each device consists of three components: a double-stem spacer made of third-generation silicone elastomer according to ISO 14949 [24] requirements and two metal rings (called “Grommet”), one for each bone segment of the joint. The double-stem spacer is a one-piece device in which the following elements can be identified: the hinge, the central part of the spacer, which, from an anatomical point of view, replaces the osteo-cartilaginous heads of the metatarsophalangeal joint of the first ray of the foot; the proximal stem, characterized by a geometry having a rectangular cross-section for better anatomical adaptation to the metatarsal endomidollar canal; and the distal stem, characterized by a geometry having a trapezoidal cross-section for better anatomical adaptation to the shape of the endomidollar canal of the phalanx. The stems are inclined to each other with the aim of maintaining the physiological position of the big toe at rest while avoiding stresses in the silicone elastomer. The Grommets are made of titanium alloy according to EN ISO 5832-3 [25] requirements and are specially shaped to be placed at the phalangeal and metatarsal intramedullary canal. Their use is to avoid direct contact between the bone tissue and the silicone material of the spacer and to improve the stability of the spacer within the medullary canals.

Patients underwent monitoring and data collection at predefined intervals from the time of implantation (T0) to two years later (T24). A questionnaire was drafted to be submitted to the patient during the visits based on the American Orthopaedic Foot and Ankle Society-Hallux Metatarsophalangeal-Interphalangeal Scale (AOFAS-HMI) [26] and Visual Analogue Scale (VAS) [27]. These visits were conducted between January 2018 and November 2024.

All patients fulfilled the inclusion criteria: (1) age between 18 and 80 years; (2) skeletally mature patient; (3) the patient suffers from severe pain and disability requiring intervention based on physical examination and history; (4) the patient failed conservative treatment; (5) the patient is willing and able to cooperate with required postoperative therapy; and (6) the patient meets at least one of the indications of osteoarthritis, post-traumatic arthritis. Exclusion criteria included: (1) the patient is unwilling or unable to give consent or comply with the follow-up schedule; (2) the patient is a known drug addict, a known alcoholic or anyone who is unable to understand what is required; (3) a pregnant or nursing patient; and (4) the patient meets at least one of the contraindications: inadequate muscle–tendon and skin system, unsuitability of the neurovascular system, bone demineralization at a significant stage, inadequate bone conformation, ongoing infection, active sepsis, psychological state of the patient such that implantation is not recommended.

From the initial cohort of 59 patients, 22 patients (37%) were lost during follow-up: 10 moved far from the clinic, 7 could not be contacted and 5 were unwilling to return for the visit.

Data from the remaining 37 patients (62.7%), corresponding to 37 arthroplasties, were then analyzed. The patient group included 23 women and 14 men with an average age of 59.8 years (range, 30–81) at the time of the operation (Table 1). Almost all of the patients were operated for hallux rigidus (81.1%) at stage III or IV according to the Coughlin and Shurnas classification. A few cases of hallux valgus rigidus (10.8%) and a few cases of a painful or unstable joint following previous surgery (5.4%) are reported. One patient was operated on following a stiff hallux due to gout (2.7%).

### 2.2. Operative Technique, Postoperative Care and Follow-Up

**Figure 1:**
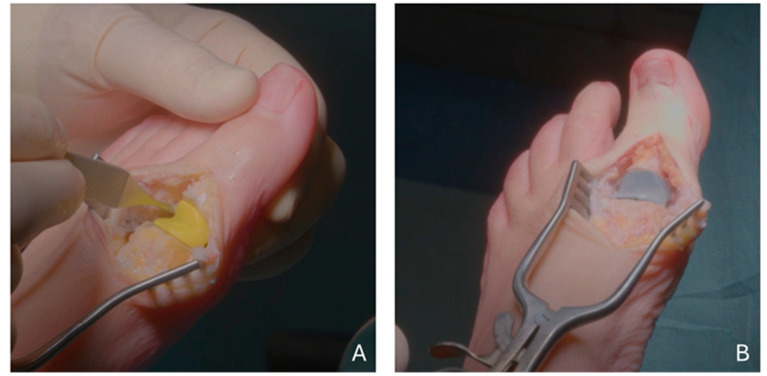
Intraoperative pictures illustrating (**A**) mobility and stability check through the trial spacer and (**B**) implant in final position before closure.

The same surgeon SF operated on all patients using the same surgical technique. A dorsal incision medially to the long extensor tendon of the big toe and a full thickness capsulotomy were made. Then, the deformed and hyperostotic portions were removed by means of rongeur and saw. According to the patient’s anatomy the corresponding size of the device to be implanted was assessed and accordingly the cutting guide was selected from the three available sizes. In order to be able to proceed with an adequate and safe bone resection, it is important that the guide fits with the bony portions as well as possible. For this purpose, it may be necessary to prepare the bone surfaces of the dorsal and medial area of the joint with a saw in order to make them flat and ensure maximum stability between the guide and bone. Subsequently, economical resection of the metatarsal and phalangeal joint surfaces was made taking care not to injure the tendon of the flexor brevis in the plantar area. The resected portions are removed, and the resulting surfaces are verified to be smooth and evenly angled. Using a tip, the metatarsal and phalangeal medullary canals are located and, using the appropriate rasps, mount directly on the handle, for manual use, or on the adapter for motorized use, the canals were prepared to accommodate the implant stems and grommets. Once the channels had been prepared, the trial spacer was inserted to check the perfect adherence to the surface of the resected bone planes (Figure 1). Mobility and stability of the implant were then checked. Once the final size was established, the ‘press fit’ grommets were inserted using the appropriate impactors. The grommets should be brought into contact with the resected bone surface. If there was a bony formation inside the canal that interfered with the taproots of the spacer, it was removed with a Volkmann’s spoon. Finally, the device was inserted into the prepared area, paying attention to insert first the metatarsal stem and then the phalangeal stem. Both the mobility and stability of the device were checked by flexion-extension movements of the big toe. If it was deemed necessary, an intraoperative fluoroscopic check was performed to assess positioning of the grommet and the implant itself. The capsule, subcutaneous and skin planes were sutured, and an elasto-compressive bandage was applied.

The implant used is available in 5 sizes for both left and right laterality (20L, 30L, 35L, 40L, 50L, 20R, 30R, 35R, 40R and 50R). The size 30 implant was used in 14 patients (37.8%), followed by the size 35 in 10 patients (27%) and then the 40 in 8 patients (21.6%), all equally divided between right and left laterality. Finally, three 20R sizes (8.10%) and two 20L sizes (5.4%) were implanted. Grommets were used on both metatarsal and phalangeal sides in all patients except 3 patients. A total of 2 patients had only the proximal grommet implanted due to the small size of the first phalanx; 1 patient had no grommet implanted due to poor press fit for small size of the bone portions.

Patients are advised to walk immediately after surgery without crutches, using only their postoperative shoes. They are also advised to practice passive foot movement in dorsiflexion and plantarflexion for 5–10 min every hour during the first postoperative month, if tolerated. All patients returned for routine visits at 1 month, 6 months, 1 year and 2 years. During the visit a surgeon assesses the patient’s situation, improvements or worsening at an interval since the operation. To do this, the surgeon used the AOFAS-HMI scoring system [26]. In addition, the patient’s perceived pain is annotated through the VAS scale score [27].

### 2.3. Data Analysis

The collected data were statistically analyzed using R 4.2.2 software (R Foundation for Statistical Computing, Vienna, Austria). Descriptive statistics were used to investigate the data.

## 3. Results

**Figure 2:**
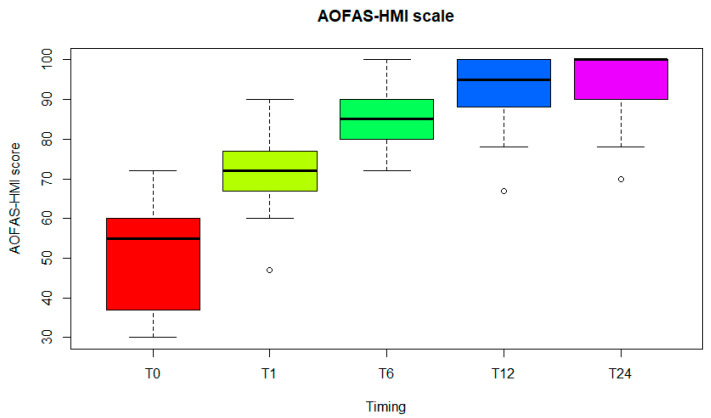
AOFAS-HMI scale at T0: the time of implantation, T1: one month later, T6: six months later, T12: one year later and T24: two years later.

**Figure 3:**
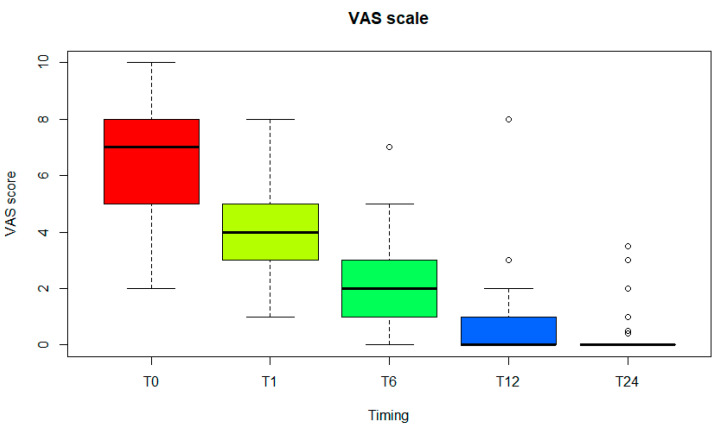
VAS scale at T0: the time of implantation, T1: one month later, T6: six months later, T12: one year later and T24: two years later.

**Figure 4:**
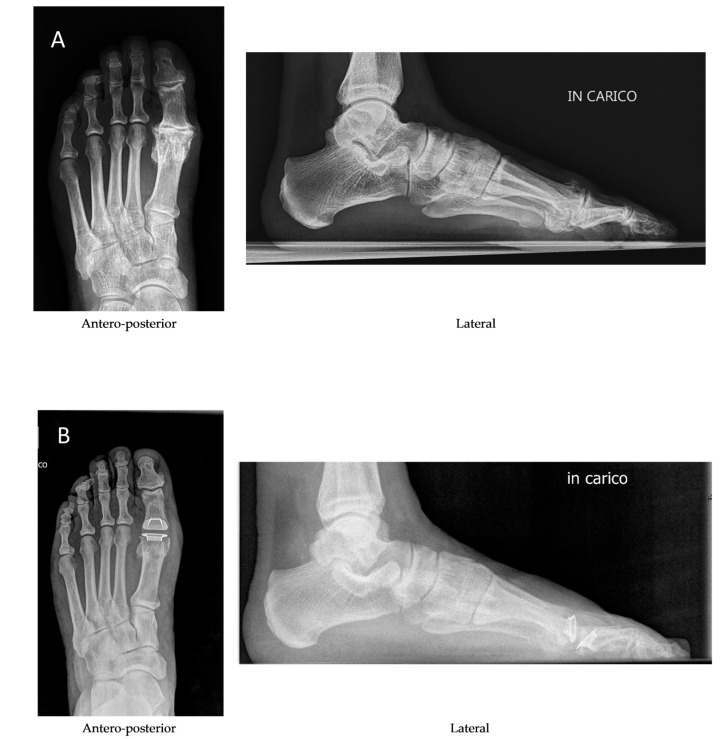
(**A**) Preoperative and (**B**) postoperative X-ray weight bearing.

The 37 patients (37 ft) were evaluated at a follow-up of 2 years. There were no intraoperative complications and no postoperative complications. The mean overall AOFAS-HMI score (Figure 2) improved from 51.0 ± 12.2 (median, 55; range, 30–72) preoperatively to 94.6 ± 7.6 (median, 100; range, 70–100) at the final follow-up (Table 2). Hallux pain was ‘none’ in 29 ft (78.4%), ‘mild’ occasional’ in 6 ft (16.2%) and ‘moderate daily’ in 2 ft (5.4%). Activity level was ‘without limitation’ for 36 ft (97.3%) and ‘with limitation of recreational activities’ for 1 ft (2.7%). Fashionable or conventional shoes were worn for 33 ft (89.2%) while comfort footwear or shoe inserts were required for 4 ft (10.8%). Stability was satisfactory in all feet (100%), and symptomatic no callous formation was observed. Alignment was ‘good: hallux well aligned’ in 33 ft (89.2%), ‘fair: some degree of hallux malalignment, asymptomatic’ in 4 ft (10.8%) and ‘poor: obvious symptomatic malalignment’ in none (0%). There were no revisions or reoperations at a minimum follow-up of two years. In addition, pain was assessed using the VAS scale (Figure 3): the mean overall score improved from 7.1.0 ± 1.9 (median, 7 range, 2–10) preoperatively to 0.4 ± 0.80 (median, 0; range, 0–3.5) at the final follow-up. An X-ray image before and after the operation is shown in Figure 4. No differences were found between patients who received both grommets and those who received one and none.

## 4. Discussion

The aim of the present study was to present the short-term results of a series of 37 patients (37 feet) who received an arthroplasty of the MTP1 joint using a third-generation double-stemmed spacer made of high-performance silicone.

At a two-year follow-up, the mean AOFAS-HMI score was 94.6 while the mean VAS score was 0.4. These results (Table 3) were compared with the available literature from the past 15 years reporting scores obtained in patients undergoing the same arthroplasty using third-generation silicone devices [8,19,20,28,29,30,31,32]. The AOFAS-HMI score obtained in the present study appears to be in line with the scores found in the literature. Santandrea et al. [20] present a study with a mean follow-up of 20 months, comparable to the one described in this article. A total of 41 cases of MTP1 arthroplasty with Primus FGT silicone implant are analyzed. The mean overall AOFAS-HMI score improved from 44.0 (range, 24–65) preoperatively to 89.0 (range, 77–100) at the final follow-up.

Some major complications associated with such surgeries, such as superficial and deep infections, bone resorption, osteolysis, symptomatic callus formation, stiffness, wound dehiscence and implant fracture, are found in the literature [3,19,20,32]. The major complication turns out to be metatarsalgia, which, however, is common to all procedures for the treatment of hallux rigidus and is not due only to the arthroplasty procedure using a silicone device [20,32,33,34,35]. Patients in this study who received Silktoe^TM^ implantation showed none of the described complications 2 years after implantation. This study demonstrates a device survival at 2 years after surgery of 100%, almost 90% of the patients analyzed are wearing fashionable shoes and none have a symptomatic bunion. The survival of these devices (Table 3), as shown in the literature, is significantly improved when compared to first- or second-generation devices [36]. The survival rate and the absence of some complications can be attributed to the implantation of grommets that prevent abrasive friction and damage to the silicone component [3,19].

The current study also demonstrates that arthroplasty surgery leads to a decrease in the patient’s perceived pain as the VAS score decreased from 7.1 to 0.4 in accordance with Santandrea et al. [20] who, at 20 months of follow-up, recorded a decrease in VAS score from 8 to 0.8.

Park et al. [37] carried out a meta-analysis of comparative studies to determine whether there is a significant difference between silicone implant arthroplasty and arthrodesis for the treatment of hallux rigidus. The purpose was to determine whether there was a significant difference between the two methods in terms of clinical scores, pain, patient satisfaction, revision surgery and complications. The results show that the VAS scale score is lower in the arthrodesis group than in the arthroplasty group. In contrast, no significant differences were found in the AOFAS-HMI score nor in the revision rate or complication rate. Park et al. confirm that there are no significantly imported differences between the two groups [37]. Other studies [18,38,39] have reported equivalent survival rates for other devices used for MTP1 arthroplasty, but the associated follow-ups are shorter and AOFAS-HMI scores appear to be slightly lower. Although the gold standard indicated to date is still arthrodesis, these results provide evidence to support arthroplasty of the MTP1 joint using a third-generation double-stemmed spacer made of high-performance silicone in the treatment of advanced hallux rigidus in patients who wish to maintain joint motion [2].

Fieschi et al. in their study, which was conducted to demonstrate the safety and performance of the Primus FGT silicone implant device [19], conclude that the good results obtained can be attributed to four features of the device:

- The use of grommets in most feet prevented abrasive friction and damage to the double-stemmed silicone component [28];
- The high-performance silicone material prevented reactive synovitis and lymphadenopathy typically associated with hinged silicone components [7,18];
- The angulation of the silicone collars and adjustment of surgical technique to avoid damaging the flexor hallucis brevis tendon grants superior joint mobility and a more natural gait [31,40];
- The sagittal angulation of the metatarsal and phalangeal stems reproduces the alignment of the toe in a neutral position and therefore maintains natural muscle and tendon tensions at rest and while walking.

These features can also be found in the Silktoe^TM^ device; indeed, there is the presence of the grommets, which prevents abrasive friction and damage to the silicone component; it is made of high-performance silicone that decreases the risk of synovitis and lymphadenopathy; the surgical technique used and the angulation of the stems do not damage the flexor tendon of the big toe thus ensuring superior joint mobility and a more natural gait; and the sagittal angulation of the stems reproduces the alignment of the big toe and maintains the natural muscular and tendon tensions both at rest and during walking.

Although arthrodesis is still considered the gold standard for severe cases of hallux rigidus, this technique results in a total loss of joint mobility. Joint arthroplasty, on the other hand, is correlated with a more uncertain outcome but allows patients to regain mobility and reduce pain. Among possible arthroplasties, prosthetic arthroplasty with a double-stem silicone implant appears to be a viable option as reported in the studies discussed in this article. Specifically, double-stemmed silicone prostheses for MTP arthroplasty have been on the market for many years, and the currently available devices, including the Silktoe^TM^, are termed “third-generation”, in that they have a modified geometry compared with the first proposed models, which allows for a better fit of the device to the anatomical spaces. In addition, these prostheses are made of a high-performance silicone material that is more resistant to wear and shear, making the prosthesis less prone to degradation during its useful life. Finally, the use of grommets protects the silicone spacer from abrasion, helping to improve its strength.

Our study has strengths and limitations that need to be emphasized. This study was conducted on patients operated on by the same surgeon with the same surgical technique, and a single evaluation protocol was used for each patient at each visit. The limitations of this study are the small patient sample size of 37, smaller than the average number of patients included in the other studies analyzed; the percentage of patients lost during follow-up (37%) as some did not return for visits and others resided far from the clinic; and the absence of long-term follow-up data; indeed, the data collected in this study are at two years of follow-up. In addition, a non-validated assessment tool (the AOFAS-HMI score) was utilized [41,42]; however, the AOFAS scale has been widely used as a standard rating scale, and many studies report AOFAS scores to support their conclusions regarding the outcome of surgery [41].

## 5. Conclusions

The data from this study presented excellent short-term results for patients who received an arthroplasty of the MTP1 joint using a third-generation double-stemmed spacer made of high-performance silicone. All patients who received the Silktoe^TM^ implant for hallux rigidus, hallux rigidus plus valgus, painful or unstable joint following previous surgery or hallux rigidus due gout were satisfied two years after implantation. AOFAS-HMI scores of 94.6 and VAS scores of 0.4 were obtained at a two-year follow-up. These values were in line with results reported in the literature for similar devices with a two-year follow-up: AOFAS-HMI scores of 89 and VAS score of 0.8. The overall clinical results confirm the findings in the literature, which have demonstrated the efficacy of this type of double-stemmed silicone implant for MTP-1 arthroplasty, although longer-term follow-up is needed to confirm their safety and performance.

## Figures and Tables

**Table 1:** Patient characteristics.

	MTP1 Arthroplasty n = 37
Age	59.8 ± 10.2 (30–81)
Women	23 (62.1%)
Indications	
Hallux rigidus	30 (81.1%)
Hallux rigidus plus valgus	4 (10.8%)
Painful or unstable joint following previous surgery	2 (5.4%)
Hallux rigidus due to gout	1 (2.7%)

**Table 2:** 2-year follow-up AOFAS-HMI score.

	Mean ± SD	Median	Range
Pain	37.3 ± 5.6	40	20–40
Function			
Activity limitation	9.9 ± 0.5	10	7–10
Footwear requirements	9.5 ± 1.6	10	5–10
MPT joint motion	8.7 ± 2.3	10	5–10
IP joint motion	5 ± 0.0	5	5–5
MPT-IP stability in all directions	5 ± 0.0	5	5–5
Callus related to hallux MPT-IP	5 ± 0.0	5	5–5
Alignment	14.2 ± 2.2	15	8–15
Total	94.6 ± 7.6	100	70–100

**Table 3:** Data from other studies in the last 15 years.

Reference	Year	Follow-Up Period (Years)	Number of Arthroplasties Performed	Silicone Spacer Model	AOFAS-HMI ^a^ (Mean Score)	Survival ^a^ (%)	VAS ^a^ (Score)
This study	2025	2.0	37	Silktoe	94.6	100	0.4
[8]	2010	9.0	59	Swanson	83.0	85.9	n.a.
[19]	2015	5.0	70	Primus	88.9	100	n.a.
[20]	2019	2.0	41	Primus	89.0	n.a.	0.8
[28]	2012	19.0	42	Swanson	n.a.	97.2	n.a.
[29]	2012	8.5	108	n.a.	77.5	97.1	2.3 ^b^
[30]	2011	7.2	92	n.a.	82.4	95.2	n.a.
[31]	2013	5.5	70	Primus	87.4	94.9	2.5 ^b^
[32]	2023	4.0	112	Swanson	n.a	99.1	1.5

^a^ Value at final follow-up. ^b^ Value calculated through the data available in the reference studies.

## Data Availability

The data presented in this study are available upon request from the corresponding author.

## Abbreviations

The following abbreviations are used in this manuscript:

MTP1	first metatarsophalangeal
PVA	polyvinyl alcohol
AOFAS-HMI	American Orthopaedic Foot and Ankle Society-Hallux Metatarsophalangeal-Interphalangeal Scale
VAS	Visual Analogue Scale
